# Exposure to Non-Extreme Solar UV Daylight: Spectral Characterization, Effects on Skin and Photoprotection

**DOI:** 10.3390/ijms16010068

**Published:** 2014-12-23

**Authors:** Claire Marionnet, Caroline Tricaud, Françoise Bernerd

**Affiliations:** 1 L’Oréal Research and Innovation, 1 avenue Eugène Schueller, 93601 Aulnay-sous-Bois, France; E-Mail: cmarionnet@rd.loreal.com; 2 L’Oréal Research and Innovation, 188-200 rue Paul Hochart, 94550 Chevilly-Larue, France; E-Mail: ctricaud@rd.loreal.com

**Keywords:** ultraviolet, skin, UV daylight, daily ultraviolet radiation, solar exposure, human skin, reconstructed skin, photoprotection, sunscreen

## Abstract

The link between chronic sun exposure of human skin and harmful clinical consequences such as photo-aging and skin cancers is now indisputable. These effects are mostly due to ultraviolet (UV) rays (UVA, 320–400 nm and UVB, 280–320 nm). The UVA/UVB ratio can vary with latitude, season, hour, meteorology and ozone layer, leading to different exposure conditions. Zenithal sun exposure (for example on a beach around noon under a clear sky) can rapidly induce visible and well-characterized clinical consequences such as sunburn, predominantly induced by UVB. However, a limited part of the global population is exposed daily to such intense irradiance and until recently little attention has been paid to solar exposure that does not induce any short term clinical impact. This paper will review different studies on non-extreme daily UV exposures with: (1) the characterization and the definition of the standard UV daylight and its simulation in the laboratory; (2) description of the biological and clinical effects of such UV exposure in an *in vitro* reconstructed human skin model and in human skin *in vivo*, emphasizing the contribution of UVA rays and (3) analysis of photoprotection approaches dedicated to prevent the harmful impact of such UV exposure.

## 1. Introduction

The skin is the most external part of the body and forms a physical barrier to the environment, providing protection against microorganisms, ultraviolet radiation, toxic agents or mechanical insults. Human skin includes three main structures: the epidermis, the dermis and subcutis. The epidermis is the external layer and is mostly composed of keratinocytes going through a vertical differentiation process, forming a stratified squamous epithelium. The final steps of keratinocyte differentiation lead to the formation of the stratum corneum, the most external barrier against environmental aggressions. The epidermis also includes: melanocytes responsible for the production of melanin pigments, Langerhans cells as antigen presenting cells, and Merkel cells interacting with nerve endings. The dermis is the area of supportive connective tissue between the epidermis and the underlying subcutis. It is a fibrous and elastic tissue that gives the skin its flexibility and strength. It contains appendages such as sweat glands and hair roots and also blood and lymph vessels. The dermis is made up of fibroblasts, which produce extracellular matrix proteins like collagens, elastin and structural proteoglycans, and also includes immune cells such as mast cells and macrophages. The subcutis is the layer of loose connective tissue and fat beneath the dermis.

Solar ultraviolet (UV) exposure is one of the most important environmental factors affecting skin physiology. Exposure of human skin to solar UV rays can lead to short and long term consequences including erythema (or sunburn reaction), photo-aging, photo-immunosuppression and skin cancers.

Solar UV rays that reach the Earth’s surface are a combination of UVB (290–320 nm) and UVA (320–400 nm). The latter comprise UVA2 or shortwave UVA (320–340 nm) and UVA1 or longwave UVA (340–400 nm). Although UVB rays display beneficial effects such as production of several antimicrobial peptides and previtamin D, they are more energetic than UVA rays, can directly damage the DNA of epidermal cells and induce sunburn reaction. In the long term, they are major contributors of photo-carcinogenesis. In turn, UVA rays penetrate deeper within skin and are mostly responsible for the generation of reactive oxygen species (ROS) and, to a lesser extent than UVB rays, can also generate DNA damage [1]. They can reach the deep dermis, induce dermal damage and, in the long term, are mostly involved in skin photo-aging [2,3,4]. Both UVA and UVB have been shown to be responsible for pigmentation, photo-immunosuppression, photo-aging, and photo-carcinogenesis [5,6].

UVA represents the vast majority of UV received on Earth (around 95%), but the UVA/UVB ratio varies according to geo-orbital and environmental factors. Geo-orbital factors include latitude, time of the year (season) and hour of the day. The solar elevation angle (SEA), the angle between the horizon and the sun, greatly influences UV irradiance: the higher the sun, the greater the UVB content. Environmental factors affecting UV irradiance include clouds and thickness of the ozone layer, which greatly influence the UVB amount reaching ground level, as well as pollutants, aerosols or the reflection of UV rays from ground [7,8,9,10]. Due to their energetic properties, UVA are less affected by these geo orbital and environmental factors and vary to a lesser extent than UVB. Therefore the UVA/ UVB ratio is highly dependent on all of the factors cited above [11,12]. Hence, different types of sun exposure conditions can be encountered.

In order to study the impact of exposure to solar UV and to determine which kind of photoprotection would be appropriate to avoid or alleviate its damaging consequences, reference spectra (standard spectra) were determined by reliable modeling using extraterrestrial data. The spectral irradiances obtained by these methods were in agreement with those obtained by measurement at ground level. It is then possible to calculate the levels and characteristics of solar spectral irradiance for any geographical site using meteorological and atmospheric parameters applied in reliable modeling formulas [7].

Two main types of exposure have been defined and can be simulated in the laboratory. The first one is the most described and represents an extreme type of exposure under a zenithal sun (solar standard spectra) leading to rapid clinical consequences. This intense exposure, its acute and chronic consequences (e.g., erythema, DNA damage and mutations, photo-immunosuppression, photo-cancers)—most of them being attributable to highly energetic UVB rays-, and their prevention have been widely studied [13,14].

However, a limited part of the global population is exposed daily to such type of sun exposure. Until recently, little attention has been paid to less extreme conditions of exposure that do not lead to any short term visible clinical impact, although a body of information now tends to prove their involvement in cutaneous long term consequences [15]. Thus, another type of sun exposure representing a non-extreme exposure, under a non-zenithal sun, had to be defined in order to better assess its impact on human skin. In this review the characterization of such a solar exposure, the standard UV daylight spectrum, and its simulation in the laboratory will be presented and compared to zenithal solar spectra. The biological and clinical impact of this type of solar UV exposure will then be detailed, as well as the photoprotection strategies for such exposure.

## 2. Conditions of Solar Exposure

In this part the two main types of solar exposure (extreme and non-extreme) will be presented and compared.

### 2.1. Solar Standard Spectra/Zenithal Solar Spectra

The research community has defined standard spectra that mimic exposure conditions including summer global sunlight (diffuse and direct sunlight), under a clear sky and a SEA greater than 80° corresponding to a quasi-zenithal sun irradiance [16,17,18]. The standard spectra exhibit a UVA/UVB irradiance ratio typically lower than 18 [13,19,20]. Corresponding exposure conditions occur in the summer, around noon, at low latitudes, and with a clear sky and represent the “worst” case scenario for human skin. Such type of exposure conditions can lead to erythema, predominantly induced by UVB. This reaction happens in a few minutes or hours, depending on the UV dose received and individual phototype; in fair skin types, sunlight may induce a transient flush of erythema during or immediately after exposure, while a delayed erythemal response is common in all skin phototypes, and peaks between 6–24 h [21].

**Figure 1:**
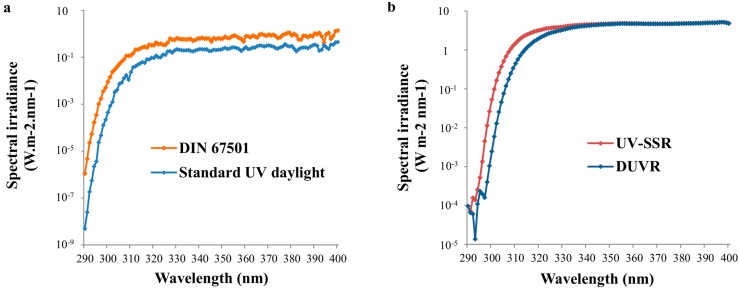
Spectral irradiance of solar spectra and their associated simulated solar spectra. (**a**) Solar spectral UV irradiance of DIN 67501 standard spectrum is representative of a spectrum given by zenithal sun [24] and standard UV daylight is representative of non-extreme solar exposure (SEA < 45) [25]; (**b**) UV-SSR and DUVR spectra are solar simulations of standard solar spectrum and standard UV daylight respectively. The irradiance axis is in logarithmic scale. Note the highest proportion of UVB (290–320 nm) and short UVA (320–340 nm) included in the DIN 67501 standard spectrum compared to the standard UV daylight spectrum. It is also the case for the corresponding simulated spectra.

*In vivo* and *in vitro* experiments have historically used various UV sources. The quality of delivered UV spectrum is a critical point and can drastically influence the physiological relevance of the data. To reproduce the standard spectra in the laboratory, it is now well established that xenon-arc solar simulators equipped with the appropriate filters are the most accurate devices [22]. Using such solar simulators, an irradiance spectrum called “UV-solar simulated radiation” (UV-SSR) including UVA and UVB wavelengths, with a UVA/UVB ratio close to 10 can be obtained. This UV-SSR spectrum is now used extensively in most of the photobiology studies using solar simulators and reproduces summer zenithal sunlight with a high UVB erythemogenic spectral portion (Figure 1). Although the irradiance of solar simulators is usually higher than that of natural sun, the minimal erythemal doses (MED)—for a given skin color phototype—have been shown to be comparable to those found in outdoor situations [23].

However a limited part of the world population is exposed daily to such intense conditions, especially in temperate latitudes but also in sunnier regions where people try to avoid uncomfortable and damaging extreme solar exposure conditions (heat and sunburn). In fact people are more often exposed to sunlight that generates no immediate and obvious clinical damage. Accordingly, until recently, little attention has been paid to such daily conditions of UV exposure although several studies using acute or repeated sub-erythemal doses of UVA or UV-SSR have revealed biological effects in human skin, including for instance DNA damage, oxidative stress, dermal changes photo-immunosuppression or pigmentation [26,27,28,29,30,31] (for exhaustive reviews, see [15,32]).

### 2.2. Standard Spectra Representing Daily Solar UV Exposure Conditions

It became interesting to determine standard spectra that represent daily exposure conditions, assuming that the biological effects obtained with such sources would be representative of those obtained under realistic average solar exposure conditions. This type of solar exposure had to exclude conditions that are more likely to induce erythema. Hence, it was considered that a solar exposure in the condition of SEA lower than 45° was not likely to lead to erythema [33].

Air mass 2 spectral irradiance is a global solar spectral irradiance corresponding to a 27.6° SEA and can be considered as representative of non-extreme exposure condition. However no spectral data for wavelengths below 300 nm were provided [7]. This spectrum has not been used in human photobiology studies.

Another relevant standard UV irradiance representing non-extreme daily exposure conditions was defined by Christiaens *et* *al*. [25] and was called “standard UV daylight” spectrum. The authors used radiation transfer calculations that were based on an updated version of the model described by Frederick and Lubin (interaction of solar UV radiation with the Earth-atmosphere system, using satellite-based solar backscattered UV measurements and a theoretical model) [34]. Spectral irradiance received at ground level by a horizontal surface was calculated over the 290–450 nm wavelengths (1 nm intervals) at a specified latitude, date and local time. Calculations were performed for latitudes from 60° South to 60° North at intervals of 10°, for one day in the middle of each month of the year, from sunrise to sunset but limited to a SEA lower than 45°. Six-year averages of column ozone values, for each latitude and months were used. An average irradiance spectrum was calculated from the 2558 obtained spectra (Figure 1a). This “standard UV daylight” spectrum exhibited an average ratio of UVA/UVB irradiance values of 27.3 ± 0.2.

It was estimated that a UV spectrum with a UVA/UVB irradiance ratio comprised between 23 and 32 was representative of UV daylight spectrum. Calculation of meteorological doses of UV received at ground level on 15 April allowed the estimation of the proportion of UV daylight in the total UV received. For latitudes distant from the equator, the proportion of UV daylight can reach more than half of the total UV received, for 15 April (Table 1).

**Table 1:** Examples of worldwide doses of Daily UV radiation (DUVR) received on 15 April. DUVR corresponds to the UV spectrum with a UVA/UVB ratio comprised between 23 and 32, corresponding to a SEA lower than 45°, for almost all the latitudes. Data were calculated from Christiaens *et al*. [25].

City	Country	Latitude (Decimal Degrees)	UV Dose (J/cm^2^)	UV Daylight Dose (J/cm^2^)	UV Daylight Proportion (%)
Oslo	Norway	59.9	112.45	57.97	52%
Copenhagen	Denmark	55.7	122.38	64.35	53%
Moscow	Russia	55.8	122.77	64.6	53%
Berlin	Germany	52.5	129.64	69.03	53%
London	England	51.5	131.94	70.5	53%
Paris	France	48.9	137.59	68.31	50%
Lausanne	Switzerland	46.5	141.98	59.55	42%
Nice	France	43.7	148.12	47.33	32%
Sapporo	Japan	43.1	148.59	46.39	31%
Chicago	USA	41.9	150.48	42.63	28%
Roma	Italy	41.9	150.8	41.99	28%
New York	USA	40.7	152.93	37.75	25%
Madrid	Spain	40.4	153.46	36.69	24%
Lisbon	Portugal	38.7	156.19	34.54	22%
Tunis	Tunisia	36.8	158.92	33.73	21%
Tokyo	Japan	35.6	161.3	33.03	20%
Los Angeles	USA	34.1	163	32.52	20%
Miami	USA	25.8	172.42	25.18	15%
Mexico City	Mexico	19.4	176.82	18.3	10%
Hanoï	Vietnam	21.0	175.99	19.57	11%
Saint Lucia	West-Indies	13.9	177.25	18.05	10%
Bangkok	Thaïland	13.8	177.27	18.04	10%
Darwin	Australia	−12.5	154.57	11.08	7%
Brasilia	Brazil	−15.8	147.72	14.15	10%
Saint Denis	Reunion	−20.9	137.56	17.76	13%
Johannesburg	South Africa	−26.2	125.54	18.3	15%
Brisbane	Australia	−27.5	122.43	18.44	15%
Sydney	Australia	−33.9	106.31	22.55	21%
Cape Town	South Africa	−33.9	105.85	22.72	21%
Auckland	New Zealand	−36.5	101.5	26.7	26%
Melbourne	Australia	−37.8	95.55	26.56	28%

For laboratory studies, simulation of standard UV-daylight can be performed using the “Daily UV radiation” (DUVR) spectrum (also called “simulated UV Daylight” spectrum or “simulated UV-DL” in some studies). DUVR spectrum exhibits a UVA/UVB irradiance ratio comprised between 23 and 32 and can be delivered using a solar simulator equipped with a dichroic mirror and a WG320 filter of correct thickness (approximately 2 mm; Schott, Clichy, France) [35] (Figure 1b).

The simulation of the standard UV daylight spectrum using the DUVR spectrum enabled the characterization of clinical and biological effects induced by daily solar exposure conditions; *i.e.*, conditions of exposure that do not lead to any short term visible clinical impact.

## 3. Effects of Exposure to Daily UV Radiation

The impacts of DUVR exposures were determined in several studies using DUVR spectrum in *in vivo* clinical studies and in *in vitro* experiments.

### 3.1. Effects of DUVR in Human Skin in Vivo

In order to investigate the impact of a non-extreme solar exposure in human skin, the first clinical and biochemical study was conducted in 12 volunteers exposed to acute, and in 22 volunteers exposed to repeated sub-erythemal doses, of DUVR or to UV-SSR [36].

Individual minimal erythema doses (MED) of DUVR and of UV-SSR were determined. For skin phototypes II and III, the average MED of DUVR and UV-SSR was found to be 12 ± 2.1 and 3.4 ± 0.55 J/cm^2^, respectively.

With regards to the biological effects induced by acute exposure to DUVR, most significant changes were obtained using 1 or 1.5 MED of DUVR, with the formation of sunburn cells (SBC), the accumulation of nuclear p53, thymine dimers, fibroblast apoptosis, a decrease in number and size of Langerhans cells, as well as an increased number of melanocytes. UV-SSR was more efficient than DUVR to induce SBC and p53 accumulation, in agreement with the known contribution of UVB in these effects. The dose of 0.5 MED of DUVR did not lead to any significant alteration of the tested endpoints but interestingly, a linear dose-response effect of DUVR was evidenced for p53 accumulation and the induction of dermal apoptotic cells.

One single exposure to a sub-erythemal DUVR dose had no significant effect, but assuming that the harmful consequences of daily UV exposures mostly result from chronic exposure, the cumulative effects of DUVR exposure were investigated. Volunteers were submitted to 9 repeated exposures to sub-erythemal doses of DUVR (0.25, 0.5 and 0.75 MED), or to 19 repeated exposures to 0.5 MED DUVR (Table 2). Exposure to 9 repeated sub-erythemal doses of DUVR led to significant changes in skin pigmentation, as assessed by colorimetric measurement using the Commission Internationale de l’Eclairage CIE lab 1976 color system, with L* expressing Luminance (from black to white), a* red-green component and b* yellow-blue component. The absolute values of L*, a*, b* are used to define the color of the skin. Δa*, Δb*, ΔL* are the differences between exposed and non-exposed sites of a *, b * and L * values, respectively. This exposure also led to significant changes in skin hydration, elasticity and microtopography, such as loss of skin density (Table 2). Biological alterations and damage were also observed, including an increase in the epidermal thickness, a decrease in number of Langerhans cells together with an increase of their size, urocanic acid isomerization [23], an increase in number and size of melanocytes and melanin deposition, an increase in keratinocyte proliferation, as well as SBC formation and p53 accumulation. The dermis was also affected with the induction of tenascin, a decrease in fibrillin and pro-collagen I, and a reduction of glycosaminoglycan deposition (Table 2). Importantly, most of the skin changes evidenced following 9 repeated exposures occurred at the lowest dose of 9 × 0.25 MED that did not induce any erythema reaction [36]. This 0.25 MED dose corresponds to 5% of the UV daylight dose received on a horizontal surface, during the day-time in mid-April (6:00 am–08:00 pm) in Paris, France (Table 1). Exposure to 19 repeated doses of 0.5 MED DUVR led to most of the skin changes cited above (Table 2) [36].

**Table 2:** Summary of alterations induced in human skin by repeated exposures to Daily UV radiation (DUVR) [23,36].

Parameters	DUVR Spread over 2 Weeks			DUVR Spread over 4 Weeks		
	9 × 0.25 MED	9 × 0.50 MED	9 × 0.75 MED	19 × 0.5 MED		
**Clinical Parameters**						
Pigmentation						
Δa*	+	++	+++	++		
Δb*	ns	+	++	+		
ΔL*	−	−−−	−−−	−		
Erythema	ns	+	++	+		
Hydration	−	−	−	ns		
Biomechanical properties						
Elasticity	ns	−	−	ns		
Residual deformation	ns	ns	ns	ND		
Microtopography					
Number of wrinkles	ns	ns	−	+
Coefficient of developed profile	ns	ns	−	ns
Loss of skin density (densiscore) ^§^	ND	ND	+	ND
**Biological parameters**					
Epidermis					
Histology					
Epidermal thickness	ns	ns	+	+
Langerhans cells					
Number of Langerhans cells	−	−−	−−−	−−
Size of Langerhans cells	+	++	+++	ns
Urocanic acid isomerization	+	ND	ND	ND
Melanocytes					
Number of melanocytes	+	+	+	+
Size of melanocytes	+	++	+++	+
Melanin deposition	+	++	+++	+
Proliferation					
Ki-67 + cells	+	++	+++	ns
Cellular damage					
sunburn cell formation	ns	+	+	+
p53 accumulation	ns	++	+++	+
Dermis					
Tenascin	ns	ns	++	+
Elastin	ns	ns	ns	ns
Fibrillin	ns	−	−	ND
Lyzozyme/elastin	ns	ns	ns	+
Pro-collagen I	−	−−	−−−	ns
Pro-collagen III/Pro-collagen I	ns	ns	+	ns
Glycosaminoglycan deposition	−	−	−	−−

Δa*, Δb*, ΔL* are the differences between exposed and non-exposed sites of a*, b* and L* values, respectively; ND, not determined; ns, not significant compared to non-exposed site; **+**, significant increase compared to non-exposed site; **−**, significant decrease compared to non-exposed site; the number of + or – reflects the intensity of increase or decrease compared to non-exposed site, respectively; Twelve and 10 volunteers were enrolled for DUVR exposure spread over 2 weeks and over 4 weeks, respectively. ^§^ study conducted in 19 volunteers for densiscore measures.

These results indicate that under repeated exposures to a realistic DUVR dose that does not lead to any sunburn reaction, several significant clinical and biological skin alterations can be induced in both epidermal and dermal compartments. The study also evidenced that some biological endpoints were more sensitive to UV-SSR such as SBC formation, whereas activation of melanocytes was more sensitive to DUVR, indicating that UV spectrum is of high importance regarding the biological and clinical impacts of UV rays on skin, as shown in previous studies [37,38]

**Figure 2:**
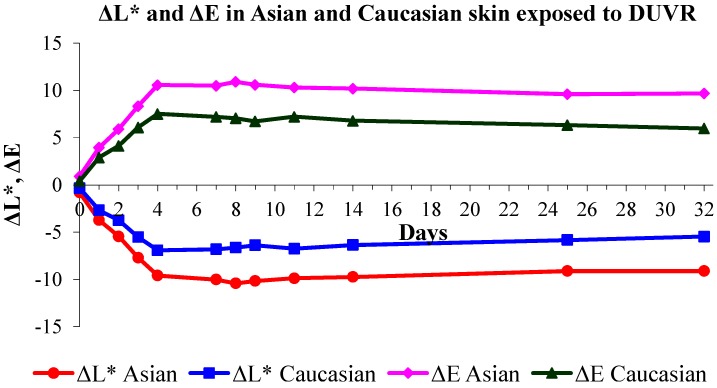
Pigmentation induced in human skin exposed to DUVR. Variation of skin pigmentation (ΔE) and luminance (ΔL*) induced by four exposures of 0.75 MED DUVR (from day 0 to day 3) in Caucasian and Asian skin. The evolution of the color of the skin expresses itself through the combination of changes of the coordinates L * a * b * as follows: ΔE = [(ΔL*)^2^ + (Δa*)^2^ + (Δb*)^2^]^1/2^, where Δa*, Δb*, ΔL* are the differences between exposed and non-exposed sites of a*, b* and L* values, respectively.

The impact of DUVR on skin pigmentation was further investigated regarding ethnic origin (Figure 2). Ten Caucasian volunteers and 8 Asian volunteers with similar constitutive pigmentation were enrolled (mean individual typologic angle (ITA°) value of 34° and 35° for Caucasian and Asian volunteers respectively, [39]). Volunteers were exposed four times to 0.75 MED DUVR daily, from day 0 to day 3. Skin color was assessed by colorimetric measurement using L*a*b* color system and by visual scoring, before each DUVR exposure and at different time points until day 32, *i.e.*, 29 days after the last DUVR exposure.

A significant increase in skin pigmentation was detected for both populations (decrease in luminance ΔL* and increase in ΔE) after DUVR exposure, from day 1 to day 32, compared to day 0 (Tukey test, *p* < 0.001). Seventy-two hours after the last DUVR exposure (day 7), pigmentation was stable and persistent (Figure 2). Results also clearly showed that pigmentation induced by DUVR was significantly higher in Asian skin compared to Caucasian skin (Tukey test, *p* < 0.05) (Figure 2). Colorimetric measurements were confirmed by visual assessment using a scale scoring from “absence of pigmentation” to “darker brown pigmentation”.

To summarize, in human skin *in vivo*, acute or repeated exposures to DUVR can modulate detectable short term clinical parameters such as pigmentation, hydration, and microtopography, and can induce biological and biophysical alterations in the dermis and the epidermis, that could be linked to long term adverse clinical effects such as photo-aging including pigmentary disorders and photo-cancers.

### 3.2. In Vitro Effects of DUVR in Reconstructed Human Skin Model

In order to better characterize the cellular and molecular impact and the early events induced by non-extreme exposure conditions, *in vitro* studies were performed using a three dimensional (3D) reconstructed human skin model composed of a dermal equivalent including living adult fibroblasts covered by a fully differentiated epidermis. The 3D architecture of the model enables UV penetration properties, depending on wavelength, to be taken into account. The model has been shown to be a useful tool for studying the responses of fibroblasts and keratinocytes to solar UV exposure *in vitro* and can reproduce sunburn related markers and dermal damage associated with the photo-aging process [40,41].

#### 3.2.1. Biological Efficient Dose and Histologic Changes

**Figure 3:**
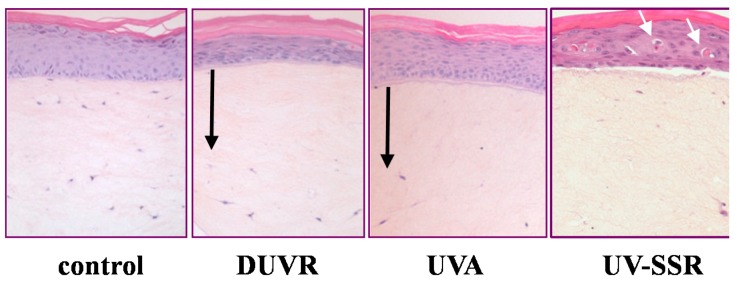
Morphological changes induced by the biologically efficient doses of DUVR (13 J/cm^2^), of UVA (25 J/cm^2^) or of UV-SSR (5.4 J/cm^2^) in reconstructed human skin [43,44,45,46]. Black arrows indicate the zone where the incidence of fibroblasts has decreased. White arrows indicate sunburn cells.

Since the MED determination could not be achieved in such *in vitro* experimental conditions, the biological efficient dose (BED) has been previously defined as the minimal dose able to induce morphological alterations after acute UV exposure [42,43]. Histological analysis of this reconstructed skin model exposed to increasing doses of DUVR established the DUVR BED at 13 J/cm^2^. At this dose, observed alterations were mostly located in the dermal compartment and were characterized by the disappearance of fibroblasts. Such changes have also been observed following exposure to UVA alone [42,43] (Figure 3). Some alterations were also detected in the epidermis. These included slight alterations in the granular layer resembling those observed after UVA exposure, as well as thinning of the epidermis and thickening of the cornified layer. Moreover, at this BED of DUVR, few sunburn cells and p53 positive keratinocytes could be detected. The histological damage induced by DUVR was correlated with the release of the well-known matrix metalloproteinase 1 (MMP-1), a photo-aging marker in the culture medium of reconstructed skin [44,45]. To summarize, the BED of 13 J/cm^2^ DUVR induced histological alterations mostly in the dermis, as observed after UVA and some alterations in the epidermis that were similar to those induced by UV-SSR or UVB (Figure 3) [46]. The lower dose of 7 J/cm^2^ DUVR was not sufficiently high to induce any of the cited histological damage. Repetitive exposures to DUVR for five consecutive days showed drastic alterations in the dermis and in the epidermis, even with the sub-BED dose of 7 J/cm^2^, attesting that chronic exposure to low DUVR dose may account for long term harmful consequences [45].

The determined BED of DUVR in a reconstructed skin model (13 J/cm^2^) corresponded to a realistic dose since it represented 20% of the daily dose of UV received in Paris on mid-April (Table 2) and was correlated with human *in vivo* data that established an average MED of 12 ± 2.1 J/cm^2^ DUVR for skin phototypes II and III [25,36].

#### 3.2.2. Modulation of Gene Expression

To further characterize DUVR induced changes, gene expression was studied in reconstructed skin exposed to DUVR using cDNA arrays and quantitative PCR. The expression of more than 200 genes related to skin biology and stress response was studied in fibroblasts and keratinocytes separately. DUVR induced the modulation of expression of numerous genes in both cell types. In the cDNA arrays profiling, the biological efficient dose of 13 J/cm^2^ DUVR induced the modulation of 27% and 31% of the genes analyzed in fibroblasts and keratinocytes respectively [47]. In the study using QPCR arrays 16% and 27% of the genes analyzed were found modulated by 12 J/cm^2^ DUVR, in fibroblasts and keratinocytes respectively [48]. These results confirmed the impact of DUVR at the surface and in deeper layers of skin, as already described in *in vivo* and *in vitro* histological analyses.

DUVR modulated genes were related to several functional families. In the epidermis, DUVR affected the expression of keratinocytes markers involved in the differentiation/proliferation balance. Several members of the epidermal differentiation complex (filaggrin, loricrin, involucrin, CRCT1, SPPR1A, SPRR1B, SPRR2A, LCE2B, LCE2D) and other differentiation markers such as corneodesmosin, calmodulin-like 5, transgutaminase 1, stratifin, serpinB2 transcripts had their expression modulated by DUVR exposure. In addition, DUVR affected expression of markers related to epidermal proliferation and markers expressed in basal keratinocytes (keratin 5, keratin 6B, Ki67 and ornithine decarboxylase 1 ODC1) [48]. These changes can be linked to *in vivo* skin surface alterations following DUVR exposure such as perturbations in hydration, skin microtopography, epidermal proliferation and thickening (Table 2, [36]).

The expression of genes encoding extracellular matrix (ECM) and dermal-epidermal components as well as proteins of ECM maturation and remodeling was also affected following DUVR exposure. For instance, while the expression of collagens and fibronectin ECM components was down-regulated, the expression of remodeling genes MMP1, MMP3 and members of the plasminogen activator system serpin1, serpinB2 and plasminogen activator tissue PLAT, was up-regulated [48]. Alterations of ECM components and homeostasis have been widely described after UV exposure [49]. These changes, especially MMPs induction and collagen synthesis and repression, represent hallmarks of the photo-aging process and the formation of solar elastosis [50,51]. These data may therefore emphasize the role of such low DUVR doses in the development of photo-aging clinical signs *in vivo* (Table 2, [36]).

Genes encoding growth factors, receptors and hormones also had their expression modulated by DUVR exposure in fibroblasts as well as in keratinocytes. In this family, the expression of Heparin-Binding EGF-like Growth Factor HBEGF, Growth Differentiation Factor 15 GDF15, Transforming Growth Factor α TGFA, granulocyte/macrophage colony-stimulating factor (GMCSF/CSF2) and Fibroblast Growth Factor 7 FGF7 (also known as Keratinocyte Growth Factor KGF) was strongly up-regulated [48]. Interestingly, FGF7 and CSF2 proteins have been shown to be positive regulators of skin pigmentation. Chronic solar exposure has been linked to pigmentary disorders [52]. The formation of actinic lentigines or “age-spots”, only found in sun-exposed anatomical sites, brings irrefutable proof of this link. Up-regulation of genes related to skin pigmentation by DUVR evidenced a contribution of such non-extreme exposures to these clinical signs [36,53,54,55].

DUVR exposure also has an impact in skin immunity related markers: it strongly increased the expression of genes encoding cytokines and inflammation markers such as interleukins (IL1B, IL6, IL8), chemokines (CCL2), ICAM1, CSF2, TNF and PTGS2 (also called COX2), confirming the immune-competence of keratinocytes and fibroblasts. In contrast, several members of the innate immunity gene family had their expression down-regulated by DUVR, such as TLR1, TLR3 or TNFSF10. Again, this data reinforced the fact that daily UV exposure may also be implicated in the UV-induced immunological response of skin [56,57].

Response to stress was particularly enriched after DUVR exposure, attesting that DUVR represents a stress for skin cells. DUVR induced expression of genes encoding heat shock proteins (HSP27, HSPA1A/HSP70, HSP90, DNAJB1/HSP40, HSPA2, HSPA5), and of genes involved in cellular response to oxidative stress (this functional family will be emphasized further in this review). UV induction of HSP has already been described and is considered to be part of a natural defense mechanism against UV exposure [58,59]. In such a context, HSP70 plays a particular role in photo-aging. HSP70 and members of the HSP70 family are induced by UVB, by UVA, and by UVA1 [60,61,62]. It was recently shown that the over-expression of HSP70 in mice led to the suppression of UV-induced skin damage and resulting inflammatory responses as well as UV-induced wrinkle formation [63,64].

#### 3.2.3. Contribution of UVA Wavelengths to DUVR Biological Effects

As UV daylight includes a high and constant proportion of UVA wavelengths, with a UVA/UVB ratio around 27, corresponding to 96.5% UVA and 3.5% UVB, the biological contribution of UVA wavelengths included in the DUVR spectrum was assessed. Accordingly, gene expression profiling using cDNA arrays was performed following exposure of *in vitro* reconstructed skins to DUVR and to UVA at their respective BED (13 J/cm^2^ DUVR and 25 J/cm^2^ UVA). In fibroblasts, the expression of 225 genes was studied. Sixty genes were modulated by UVA or DUVR. Out of them 55/60 (92%) were common to DUVR and to UVA. In keratinocytes, the expression of 241 genes was studied. The vast majority (59/74, 80%) of the modulated genes were identical in DUVR or UVA exposure conditions. These results showed that both types of exposures share biological targets therefore attesting to a strong contribution of UVA wavelengths to the DUVR biological response.

In keratinocytes, 20% of genes were specifically modulated by DUVR and not by UVA. They mostly included genes involved in the differentiation/proliferation balance, such as genes of the epidermal differentiation complex. In fibroblasts, only 3% of the analysed genes were specifically modulated by DUVR. The DUVR spectrum includes wavelength ranges from UVB and shortwave UVA (UVA2, 320–340 nm) to longwave UVA (UVA1, 340–400 nm), having different and increasing penetration properties. For this reason, keratinocytes, due to their surface location, receive photons of the whole DUVR spectrum, whereas fibroblasts, in deeper layers of the skin, are mostly exposed to UVA of the DUVR spectrum. Therefore, it may be hypothesized that the 20% of genes specifically modulated by DUVR may be attributed to UVB wavelengths included in the DUVR spectrum. In contrast, fibroblasts receive the same wavelengths from the DUVR spectrum as from the UVA spectrum resulting in the same changes in gene expression.

Altogether the results established that DUVR biological impact was mostly imputable to UVA wavelengths included in the DUVR spectrum, especially for dermal fibroblasts, located in skin depth. Photo-aging due to chronic exposure to UVA was particularly well illustrated by cases of unilateral dermatoheliosis occurring on the side of face that is chronically exposed to UVA through a glass window (e.g., truck or taxi drivers) showing skin thickening, roughness, wrinkling and laxity associated with an accumulation of elastotic material within dermis [65].

#### 3.2.4. Focus on Oxidative Stress Induced by DUVR and Characterization of the Fibroblast and Keratinocyte Response

**Figure 4:**
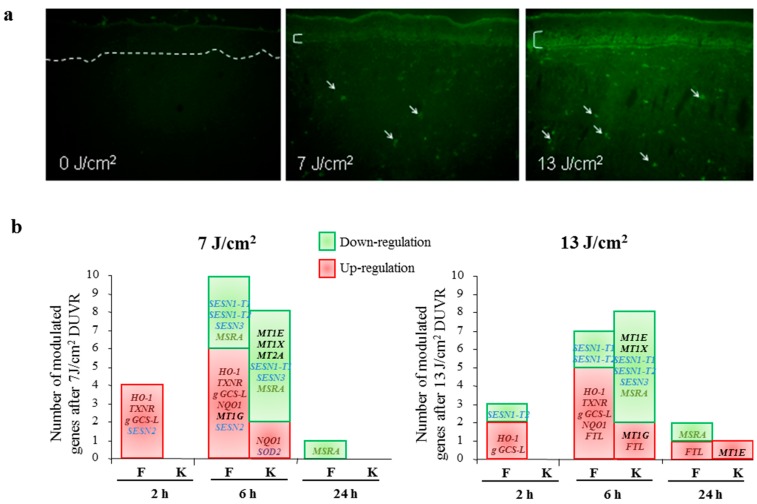
(**a**) DUVR induced ROS; (**b**) Cellular response to DUVR induced oxidative stress. Exposure to DUVR induced the modulation of the expression of genes involved in response to oxidative stress, in fibroblasts (F) and keratinocytes (K) of reconstructed human skin. White dotted line indicates dermal epidermal junction. White brackets indicate epidermal positive layer. White arrows indicate examples of positive dermal fibroblasts.

Since (1) DUVR spectrum includes a high and constant proportion of UVA wavelengths, that are well-known stimulators of ROS production and (2) it was shown that UVA wavelengths particularly contributed to DUVR biological impact, oxidative stress induced by physiological doses of DUVR was carefully studied in reconstructed human skin model [45]. DUVR induced the generation of ROS in both epidermis and dermis of reconstructed skin, with a significant dose effect (Figure 4a). Cellular response to DUVR induced oxidative stress was analyzed by studying the expression of 24 genes encoding proteins involved in oxidative stress response in fibroblasts and keratinocytes of reconstructed skin, respectively. DUVR mostly altered the expression of four gene families: target genes of the cytoprotective to oxidative and electrophilic stress NF-E2-related factor 2 (Nrf2)-pathway, sestrins that participate in the regeneration of over-oxidized peroxiredoxins, metallothioneins that scavenge ROS and metal ions, and methionine sulfoxide reductase (MSRA), that is involved in the maintenance of protein structure and function. A differential response to oxidative stress between fibroblasts and keratinocytes was revealed, with regard to kinetics, direction or levels of modulation and nature of modulated genes. In dermal fibroblasts, oxidative stress response occurred as early as two hours post exposure, with a majority of the genes up-regulated; whereas in keratinocytes gene modulations were mostly detected six hours post DUVR exposure, with a higher proportion of down-regulations (Figure 4b). Nrf2 target genes (*HO-1*, *TXNR*, *NQO1*, *gammaGCS-L*) were significantly up-regulated in dermal fibroblasts by DUVR, while in keratinocytes, only *NQO1* gene expression was significantly induced. Genes encoding metallothioneins were also differently modulated in fibroblasts and in keratinocytes, with a down-regulation by DUVR of MT1X, MT1E and MTE2A found only in keratinocytes. For the sestrin family and MSRA, the responses were quite similar between fibroblasts and keratinocytes. Most of the studied sestrins and MSRA, whose decline has been shown to be associated with aging and photo-aging, had their gene expression level decreased [66,67,68] (Figure 4b).

It was important to note that the low dose of 7 J/cm^2^ DUVR, which did not lead to any detectable histologic changes, was sufficient to generate ROS, even in deeper layers of the dermis, and to modulate the expression of genes related to several functional families described above. This reveals the insidious impact of DUVR, even in the absence of any detectable tissue damage and shows that the dermal compartment is highly susceptible to DUVR [45,47].

## 4. Photoprotection against DUVR

Since evidence has been given of damaging effects of DUVR in the whole skin, protection from UV daylight impact is paramount. In this context, sunscreen products can be evaluated. The latter are characterized by their absorption profiles, with corresponding protection factors (PF): the sun (burn) protection factor (SPF) and the UVA protection factor (UVA-PF). SPF value is determined with a standardized protocol, *in vivo* in human volunteers exposed to UV-SSR spectrum and measures the protection against erythema, which is mainly induced by UVB wavelengths. Hence SPF value does not provide information on the protection level against UVA since UVA poorly contributes to skin erythema [69]. UVA-PF can be determined *in vivo* using the Persistent Pigment Darkening (PPD) method which measures the darkening in human volunteers with phototypes III and IV when exposed to UVA spectrum. The higher the UVA-PF, the better the UVA protection [70,71]. The next paragraph will address two issues: (1) the efficiency of sunscreen products against DUVR *in vitro* and *in vivo* and (2) the relevance of the protection factors (SPF and UVA-PF) under such type of exposure.

### 4.1. Photoprotection Assessed in Vivo

Several *in vivo* studies have evaluated the photoprotection against DUVR offered by sunscreens with different absorption profiles, in different skin phototypes and ethnic origins, using several biological and clinical endpoints.

In Caucasian subjects with skin phototypes II/III, the photoprotection afforded by a broad spectrum daily-care product with a balanced UVB-UVA filtration profile (SPF 8, UVA-PF 7) was assessed against 19 repeated exposures to 0.5 MED DUVR per day over 4 weeks. This exposure regimen had been shown to induce detrimental biological effects in these volunteers (Table 2 and [36]). The use of a daily-care product with a low SPF but a well balanced UVB-UVA absorption profile inhibited or reduced most of the biological effects induced by DUVR in the dermis (decrease in GAG deposition was avoided, lysozyme to elastin ratio was not increased) and in the epidermis (the number of Langerhans cells was maintained, no noticeable increase in the number of p53 positive cells was observed, increase in the number and size of melanocytes and melanin deposition were significantly reduced). Epidermal thickening was not significantly reduced. Since the level of protection varied according to the studied endpoint, this could suggest that low doses of DUVR that are not absorbed by the sunscreen product may lead to residual damage [15].

Another study assessed the erythemal protection against DUVR, afforded by two sunscreens with comparable SPF but different levels of UVA protection (a UVB sunscreen SPF 8.6, UVA-PF 1.1 *vs.* a broadspectrum sunscreen SPF 7.0, UVA-PF 6.5), in fair-skinned sun-sensitive Caucasian subjects (skin phototypes I/II). After application of the sunscreen, the volunteers were exposed to two MED of DUVR for 13 consecutive days and erythema was measured at day 6, 8 and 13. The data show a much greater protection against cumulative erythema with the broad-spectrum sunscreen than with the UVB sunscreen. The modeling of the SPF of each sunscreen with changes in solar UVR with time of the day and latitude showed that the SPF of the broad-spectrum sunscreen is independent of latitude and time of the day, while the SPF of the UVB sunscreen varies considerably [72].

Both studies proved that an efficient protection against molecular, cellular and clinical damage induced by DUVR could be achieved in Caucasian skin with a broad-spectrum sunscreen.

In Asian skin (phototypes III, IV, V), protection against DUVR-induced pigmentation was assessed using different sunscreen formulations that included UVA + UVB absorbers, with different SPF/UVAPF ratios. The results showed that products offering a well-balanced UVB and UVA protection (*i.e.*, SPF/UVA-PF ratio lower than 3), exhibited higher protection against pigmentation than products having SPF/UVA-PF ratio higher than 3. Moreover, with the same level of SPF, a higher UVA-PF resulted in a higher protection against pigmentation in Asian skin exposed to DUVR [73].

**Figure 5:**
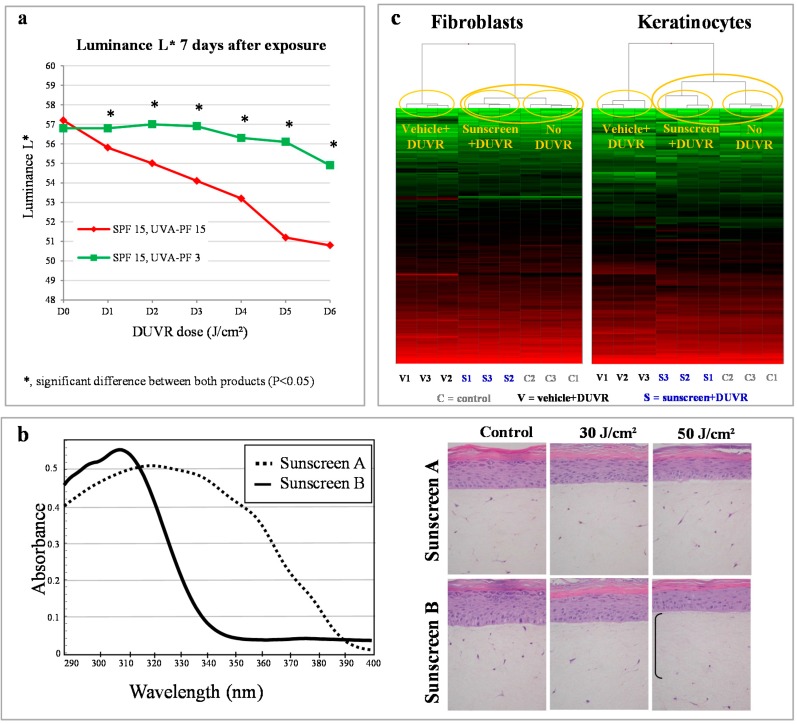
Assessment of photoprotection against DUVR *in vivo* and *in vitro*. (**a**) Luminance values seven days after exposure to increasing doses of DUVR *in vivo* in human skin protected by sunscreen with different UVA-PF [74,75]; (**b**) *In vitro*, two days after DUVR exposure, histology of reconstructed skin protected by sunscreen with different UVA-PF [44]; (**c**) Gene expression profiles in fibroblasts and keratinocytes of reconstructed skin protected or not by a broad spectrum sunscreen and exposed to DUVR [48].

In Indian and Asian skins (skin phototypes IV and V), protection against DUVR-induced pigmentation afforded by two sunscreens having the same SPF but different UVA protection factors were compared (SPF15/UVA-PF15 *vs.* SPF15/UVA-PF 3). Skin was exposed to six increasing daily doses of DUVR, with an interval of 25% between each dose. Pigmentation was assessed visually and by colorimetric measurements (L*, a*, b* parameters) 7 days after each exposure. The product with a well-balanced photoprotection against UVB and UVA (SPF 15/UVAPF 15) provided a better protection against skin darkening than the product with a low UVA protection level (SPF15/UVAPF 3) (Figure 5a) [74,75].

These *in vivo* studies show that, from light to dark skins, UVA protection is a major factor in the prevention of the DUVR induced effects, in addition to a necessary UVB protection. It should be noted that 2 mg/cm^2^ of sunscreen was applied in the *in vivo* studies. In real life, people apply a much lower amount [76], making re-application of product a safety caution.

### 4.2. Photoprotection Assessed in Vitro

To complete and support the studies of photoprotection assessed in humans *in vivo*, which are not always easy to perform, organotypic *in vitro* skin models have been shown to be useful tools [41,77].

The photoprotection afforded by sunscreen formulations to prevent histological and biochemical alterations induced by DUVR was evaluated in reconstructed human skin. Two sunscreens having similar SPF values (SPF 15) but different profiles of transmission over the UVA range were tested. A better protection of dermal fibroblasts and prevention of MMP1 production was observed when applying a well-balanced sunscreen with an efficient absorption potency in the UVA range, as compared to a sunscreen with an equivalent SPF but a lower UVA filtration (Figure 5b). Since marketed skin care products show increasing levels of SPF values, the authors wondered whether a higher SPF could compensate for a defect in UVA filtration. To address this question, 2 sunscreens were compared, one having a SPF value of 18 and a good UVA filtration profile and the other one, with a higher SPF value (SPF 27) and a poor UVA filtration profile. Results showed that even with a higher SPF value, a sunscreen product with a poor filtration in the UVA range is less effective in preventing DUVR skin damage than a well-balanced UVA-UVB sunscreen with a lower SPF value [44].

The strong photoprotection afforded by a balanced UVA-UVB sunscreen was also evidenced at the molecular level by studying gene expression. The expression of more than 200 genes involved in skin biology and stress response was studied separately in fibroblasts and keratinocytes of reconstructed skin, which was protected by applying a sunscreen product (SPF 13 and UVA-PF 10.5) or unprotected prior to DUVR exposure. In both fibroblasts and keratinocytes of reconstructed human skin, the use of sunscreen led to a significant reduction of the number of genes modulated by DUVR and a decrease in intensity of gene modulation for the residual modulated genes. This protection from DUVR induced gene modulation was particularly obvious by performing hierarchical clustering of gene expression for each experimental condition: the gene expression profile in samples protected by sunscreen and exposed to DUVR was much closer to that of unexposed samples, than that of unprotected samples exposed to DUVR (Figure 5c) [48].

In agreement with *in vivo* studies, the *in vitro* studies emphasized the importance of the use of sunscreen products filtering in an equilibrated manner UVB and UVA rays to prevent tissue, cellular, biochemical, and molecular alterations induced by exposure to solar daily UV.

## 5. Conclusions

The UV daylight spectrum represents a non-extreme sun exposure, with a SEA lower than 45°, for latitudes from 60° South to 60° North, during all the months of the year, with a UVA/UVB ratio of 27. In everyday outdoor activities, this type of exposure does not induce any visible short-term effect but may lead to long term UV-induced deleterious consequences.

Such daily sun exposure, simulated in the laboratory by the DUVR spectrum, induced *in vivo* significant clinical effects such as disturbed hydration, altered biochemical properties and microtopography of skin, and increased pigmentation. It also led to biological changes affecting the dermis—especially the composition of the extracellular matrix—and epidermis, with an impact on keratinocytes, melanocytes and Langerhans cells. *In vitro* studies evidenced that DUVR, even at low dose, induced oxidative stress, led to an alteration of the expression of genes involved in several skin and stress managing functions, in both skin compartments. The important contribution of UVA in these biological effects was also evidenced.

Efficient daily UVR protection, including UVB and UVA absorption, is necessary to avoid the sub-erythemal cumulative effects of such sun exposure. *In vitro* and *in vivo* photoprotection studies showed that, in addition to UVB protection, a sufficient UVA protection is essential to reach a significant prevention efficacy against DUVR induced damage. Moreover, the SPF value is not by itself sufficient to express the efficacy of protection against clinical, cellular and molecular effects induced by daily UV exposure. On such issues, UVA-PF appears more relevant, even if the question of an appropriate DUVR protection factor is still open.
